# 2-(Benzene­sulfonamido)pyridinium nitrate

**DOI:** 10.1107/S1600536809015670

**Published:** 2009-05-07

**Authors:** Jiang-Sheng Li, Xun Li

**Affiliations:** a School of Chemistry and Biological Engineering, Changsha University of Science & Technology, Changsha 410004, People’s Republic of China

## Abstract

In the title compound, C_11_H_11_N_2_O_2_S^+^·NO_3_^−^, the dihedral angle between the benzene and pyridinium rings is 87.59 (8)°. An intra­molecular C—H⋯O inter­action occurs in the cation. In the crystal structure, ion pairs occur, being linked by two strong N—H⋯O inter­actions, forming *R*_2_^2^(8) loops. The packing is further stabilized by weak C—H⋯O inter­actions.

## Related literature

For the synthesis, see: Li, Yang *et al.* (2008 ▶). For related structures, see: Li *et al.* (2008*a* ▶,*b* ▶). For background studies of supra­molecular chemistry involving pyridinium rings, see: Li *et al.* (2007 ▶); Li, Fan, Fan *et al.* (2008 ▶).
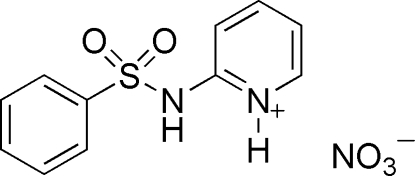


## Experimental

### 

#### Crystal data

C_11_H_11_N_2_O_2_S^+^·NO_3_^−^
*M* *_r_* = 297.29
Monoclinic, 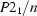

*a* = 5.3309 (11) Å
*b* = 10.067 (2) Å
*c* = 23.837 (5) Å
β = 90.44 (3)°
*V* = 1279.2 (5) Å^3^
*Z* = 4
Mo *K*α radiation
μ = 0.28 mm^−1^
*T* = 113 K
0.20 × 0.16 × 0.12 mm

#### Data collection

Rigaku Saturn CCD area-detector diffractometer
Absorption correction: multi-scan (*CrystalClear*; Rigaku, 2005 ▶) *T*_min_ = 0.947, *T*_max_ = 0.968
9882 measured reflections
2959 independent reflections
2547 reflections with *I* > 2σ(*I*)
*R*_int_ = 0.027

#### Refinement

*R*[*F* ^2^ > 2σ(*F* ^2^)] = 0.041
*wR*(*F* ^2^) = 0.107
*S* = 1.08
2959 reflections
189 parameters
H atoms treated by a mixture of independent and constrained refinement
Δρ_max_ = 0.39 e Å^−3^
Δρ_min_ = −0.50 e Å^−3^

### 

Data collection: *CrystalClear* (Rigaku, 2005 ▶); cell refinement: *CrystalClear*; data reduction: *CrystalClear*; program(s) used to solve structure: *SHELXS97* (Sheldrick, 2008 ▶); program(s) used to refine structure: *SHELXL97* (Sheldrick, 2008 ▶); molecular graphics: *SHELXTL* (Sheldrick, 2008 ▶); software used to prepare material for publication: *CrystalStructure* (Rigaku, 2005 ▶).

## Supplementary Material

Crystal structure: contains datablocks global, I. DOI: 10.1107/S1600536809015670/hb2956sup1.cif

Structure factors: contains datablocks I. DOI: 10.1107/S1600536809015670/hb2956Isup2.hkl

Additional supplementary materials:  crystallographic information; 3D view; checkCIF report

## Figures and Tables

**Table 1:** Hydrogen-bond geometry (Å, °)

*D*—H⋯*A*	*D*—H	H⋯*A*	*D*⋯*A*	*D*—H⋯*A*
N1—H1⋯O5	0.86 (2)	1.91 (2)	2.760 (2)	171 (2)
N2—H2*A*⋯O3	0.91 (2)	1.84 (2)	2.7417 (18)	173 (2)
C8—H8⋯O2	0.95	2.38	3.009 (2)	123
C3—H3⋯O5^i^	0.95	2.52	3.432 (2)	162
C10—H10⋯O4^ii^	0.95	2.53	3.193 (2)	127
C11—H11⋯O3^iii^	0.95	2.56	3.434 (2)	153
C11—H11⋯O4^iii^	0.95	2.34	3.223 (2)	154

Symmetry codes: (i) 
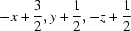
; (ii) 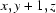; (iii) 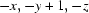.

**Fig. 1.**
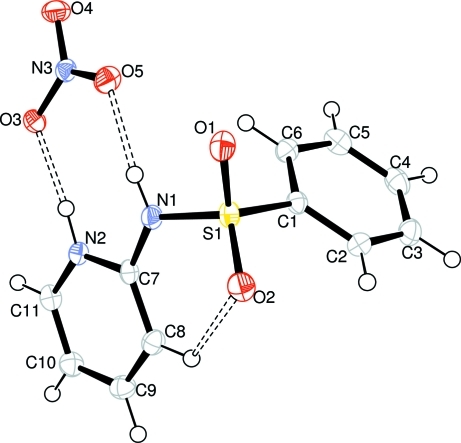
View of the molecular structure of (I) with displacement ellipsoids drawn at the 50% probability level and H atoms are shown as small spheres of arbitrary radius. Dashed lines indicates H-bonding.

## supplementary crystallographic information

### Comment

Organic pyridinium salts have been widely used as guests for construction of supramolecular complexes. As part of our ongoing studies of host–guest chemistry involving the pyridinium salts (Li *et al.*, 2007; Li, Fan, Fan *et al.*, 2008), the structure of the title compound was determined by X-ray diffraction.  For related structures, see: Li *et al.* (2008*a*,b).

The title compound, (I), consists of a pyridinium cation and a nitrate anion (Fig. 1). In the cation, the C—N distance [1.378 (2) Å] is short enough to display significant double-bond character (typical C=N = 1.34–1.38 Å), despite the presence of the strong electron-withdrawing sulfonyl group.  This could be attributed to the *ortho* N^+^ atom in the pyridinium ring. The benzene ring constructs an angle of 87.59 (8)° with the pyridinium ring.

In the crystal structure, two strong N—H···O hydrogen bonds (*R*_2_^2^(8)) link the cation and anion, and weak C—H···O interactions (Table 1) help establishing the packing as well as significant ion-dipolar interactions [N2—O2 3.020 at (*x* - 1, *y*, *z*), N2—O3 3.006, N2—O4 3.581, N2—O5 3.377 at (-*x* + 3/2, *y* + 3/2, -*z* + 1/2)].  Besides, one short intramolecular C—H···O contact also occurs in the cation.

### Experimental

The title compound was prepared according to the reported literature (Li, Yang *et al.* 2008). Colourless blocks of (I) were obtained by evaporation of a nitric acid solution of the sulfonamide.

### Refinement

The H atoms bound to C were positioned geometrically (C—H = 0.95Å) and refined as riding with *U*_iso_(H) = 1.2 *U*_eq_(C). The N—H hydrogen atoms were refined with their isotropic displacement parameters, and N—H distances are restrained to 0.86 (2) and 0.91 (2) Å, respectively.

### Crystal data

**Table:**

C_11_H_11_N_2_O_2_S^+^·NO_3_^−^	*F*(000) = 616
*M**_r_* = 297.29	*D*_x_ = 1.544 Mg m^−^^3^
Monoclinic, *P*2_1_/*n*	Mo *K*α radiation, λ = 0.71073 Å
Hall symbol: -P 2yn	Cell parameters from 3934 reflections
*a* = 5.3309 (11) Å	θ = 2.6–27.9°
*b* = 10.067 (2) Å	µ = 0.28 mm^−^^1^
*c* = 23.837 (5) Å	*T* = 113 K
β = 90.44 (3)°	Block, colourless
*V* = 1279.2 (5) Å^3^	0.20 × 0.16 × 0.12 mm
*Z* = 4	

### Data collection

**Table:**

Rigaku Saturn CCD area-detector diffractometer	2959 independent reflections
Radiation source: rotating anode	2547 reflections with *I* > 2σ(*I*)
confocal	*R*_int_ = 0.027
Detector resolution: 7.31 pixels mm^-1^	θ_max_ = 27.9°, θ_min_ = 2.7°
ω and φ scans	*h* = −6→7
Absorption correction: multi-scan (*CrystalClear*; Rigaku, 2005)	*k* = −13→10
*T*_min_ = 0.947, *T*_max_ = 0.968	*l* = −27→31
9882 measured reflections	

### Refinement

**Table:**

Refinement on *F*^2^	Primary atom site location: structure-invariant direct methods
Least-squares matrix: full	Secondary atom site location: difference Fourier map
*R*[*F*^2^ > 2σ(*F*^2^)] = 0.041	Hydrogen site location: inferred from neighbouring sites
*wR*(*F*^2^) = 0.107	H atoms treated by a mixture of independent and constrained refinement
*S* = 1.08	*w* = 1/[σ^2^(*F*_o_^2^) + (0.0535*P*)^2^ + 0.6301*P*] where *P* = (*F*_o_^2^ + 2*F*_c_^2^)/3
2959 reflections	(Δ/σ)_max_ = 0.001
189 parameters	Δρ_max_ = 0.39 e Å^−^^3^
0 restraints	Δρ_min_ = −0.50 e Å^−^^3^

### Special details

**Table:**

Geometry. All e.s.d.'s (except the e.s.d. in the dihedral angle between two l.s. planes) are estimated using the full covariance matrix. The cell e.s.d.'s are taken into account individually in the estimation of e.s.d.'s in distances, angles and torsion angles; correlations between e.s.d.'s in cell parameters are only used when they are defined by crystal symmetry. An approximate (isotropic) treatment of cell e.s.d.'s is used for estimating e.s.d.'s involving l.s. planes.
Refinement. Refinement of *F*^2^ against ALL reflections. The weighted *R*-factor *wR* and goodness of fit *S* are based on *F*^2^, conventional *R*-factors *R* are based on *F*, with *F* set to zero for negative *F*^2^. The threshold expression of *F*^2^ > σ(*F*^2^) is used only for calculating *R*-factors(gt) *etc*. and is not relevant to the choice of reflections for refinement. *R*-factors based on *F*^2^ are statistically about twice as large as those based on *F*, and *R*- factors based on ALL data will be even larger.

### Fractional atomic coordinates and isotropic or equivalent isotropic displacement parameters (Å^2^)

**Table:**

	*x*	*y*	*z*	*U*_iso_*/*U*_eq_	
S1	0.91800 (7)	0.56853 (4)	0.160276 (15)	0.01836 (13)	
O1	1.0236 (2)	0.43908 (12)	0.15364 (5)	0.0236 (3)	
O2	1.0764 (2)	0.68341 (12)	0.16025 (5)	0.0246 (3)	
N1	0.7169 (3)	0.57948 (14)	0.10753 (5)	0.0202 (3)	
N2	0.3909 (3)	0.66605 (13)	0.05567 (5)	0.0176 (3)	
C1	0.7348 (3)	0.57165 (15)	0.22140 (6)	0.0172 (3)	
C2	0.8103 (3)	0.65238 (16)	0.26571 (7)	0.0228 (3)	
H2	0.9534	0.7080	0.2624	0.027*	
C3	0.6718 (4)	0.65010 (17)	0.31498 (7)	0.0279 (4)	
H3	0.7205	0.7042	0.3459	0.033*	
C4	0.4630 (4)	0.56891 (17)	0.31892 (7)	0.0274 (4)	
H4	0.3674	0.5687	0.3524	0.033*	
C5	0.3915 (3)	0.48754 (17)	0.27435 (7)	0.0256 (4)	
H5	0.2491	0.4315	0.2777	0.031*	
C6	0.5277 (3)	0.48821 (16)	0.22514 (7)	0.0206 (3)	
H6	0.4806	0.4328	0.1945	0.025*	
C7	0.5852 (3)	0.69057 (15)	0.09074 (6)	0.0178 (3)	
C8	0.6390 (3)	0.82204 (16)	0.10529 (7)	0.0212 (3)	
H8	0.7726	0.8421	0.1305	0.025*	
C9	0.4944 (3)	0.92219 (16)	0.08236 (7)	0.0241 (4)	
H9	0.5298	1.0120	0.0917	0.029*	
C10	0.2968 (3)	0.89326 (17)	0.04570 (7)	0.0236 (3)	
H10	0.1983	0.9625	0.0298	0.028*	
C11	0.2475 (3)	0.76278 (17)	0.03302 (6)	0.0209 (3)	
H11	0.1128	0.7407	0.0084	0.025*	
H1	0.666 (4)	0.502 (2)	0.0974 (9)	0.035 (6)*	
H2A	0.347 (4)	0.580 (2)	0.0487 (9)	0.030 (6)*	
N3	0.3198 (3)	0.31725 (13)	0.05623 (5)	0.0188 (3)	
O3	0.2271 (2)	0.41507 (11)	0.02985 (5)	0.0213 (3)	
O4	0.2173 (3)	0.20711 (11)	0.05331 (5)	0.0280 (3)	
O5	0.5183 (2)	0.33211 (12)	0.08439 (5)	0.0261 (3)	

### Atomic displacement parameters (Å^2^)

**Table:**

	*U*^11^	*U*^22^	*U*^33^	*U*^12^	*U*^13^	*U*^23^
S1	0.0160 (2)	0.0236 (2)	0.01549 (19)	0.00131 (14)	−0.00045 (14)	−0.00020 (13)
O1	0.0220 (7)	0.0279 (6)	0.0210 (6)	0.0079 (5)	−0.0009 (5)	−0.0034 (4)
O2	0.0190 (6)	0.0307 (6)	0.0243 (6)	−0.0058 (5)	0.0000 (5)	0.0028 (5)
N1	0.0244 (8)	0.0196 (7)	0.0166 (6)	0.0031 (5)	−0.0039 (5)	−0.0009 (5)
N2	0.0177 (7)	0.0199 (7)	0.0153 (6)	0.0003 (5)	0.0005 (5)	−0.0015 (5)
C1	0.0167 (8)	0.0188 (7)	0.0161 (7)	0.0027 (5)	−0.0007 (5)	0.0010 (5)
C2	0.0269 (9)	0.0196 (7)	0.0219 (8)	−0.0014 (6)	−0.0019 (6)	−0.0011 (6)
C3	0.0393 (11)	0.0256 (8)	0.0187 (7)	0.0052 (7)	−0.0012 (7)	−0.0029 (6)
C4	0.0302 (10)	0.0311 (9)	0.0211 (8)	0.0080 (7)	0.0062 (7)	0.0048 (6)
C5	0.0200 (9)	0.0279 (9)	0.0290 (8)	0.0016 (6)	0.0027 (7)	0.0066 (7)
C6	0.0178 (8)	0.0215 (8)	0.0224 (7)	0.0019 (6)	−0.0030 (6)	−0.0009 (6)
C7	0.0173 (8)	0.0231 (8)	0.0131 (6)	0.0006 (6)	0.0022 (5)	0.0003 (5)
C8	0.0188 (9)	0.0240 (8)	0.0207 (7)	−0.0012 (6)	−0.0003 (6)	−0.0023 (6)
C9	0.0246 (9)	0.0210 (8)	0.0267 (8)	0.0005 (6)	0.0030 (7)	−0.0012 (6)
C10	0.0229 (9)	0.0237 (8)	0.0242 (8)	0.0037 (6)	−0.0009 (6)	0.0010 (6)
C11	0.0163 (8)	0.0272 (8)	0.0191 (7)	0.0033 (6)	0.0001 (6)	0.0014 (6)
N3	0.0201 (7)	0.0198 (6)	0.0166 (6)	0.0035 (5)	−0.0004 (5)	−0.0015 (5)
O3	0.0238 (7)	0.0204 (6)	0.0197 (5)	0.0027 (4)	−0.0040 (4)	0.0022 (4)
O4	0.0322 (8)	0.0184 (6)	0.0334 (7)	−0.0018 (5)	−0.0079 (5)	0.0002 (5)
O5	0.0240 (7)	0.0254 (6)	0.0288 (6)	0.0038 (5)	−0.0106 (5)	−0.0029 (5)

### Geometric parameters (Å, °)

**Table:**

S1—O1	1.4288 (12)	C4—H4	0.9500
S1—O2	1.4322 (12)	C5—C6	1.384 (2)
S1—N1	1.6498 (15)	C5—H5	0.9500
S1—C1	1.7607 (16)	C6—H6	0.9500
N1—C7	1.378 (2)	C7—C8	1.397 (2)
N1—H1	0.86 (2)	C8—C9	1.380 (2)
N2—C11	1.348 (2)	C8—H8	0.9500
N2—C7	1.349 (2)	C9—C10	1.394 (3)
N2—H2A	0.91 (2)	C9—H9	0.9500
C1—C2	1.390 (2)	C10—C11	1.373 (2)
C1—C6	1.391 (2)	C10—H10	0.9500
C2—C3	1.392 (2)	C11—H11	0.9500
C2—H2	0.9500	N3—O4	1.2378 (18)
C3—C4	1.385 (3)	N3—O5	1.2580 (18)
C3—H3	0.9500	N3—O3	1.2665 (17)
C4—C5	1.392 (3)		
			
O1—S1—O2	120.24 (8)	C6—C5—C4	120.12 (17)
O1—S1—N1	103.35 (7)	C6—C5—H5	119.9
O2—S1—N1	109.00 (7)	C4—C5—H5	119.9
O1—S1—C1	109.24 (7)	C5—C6—C1	118.60 (15)
O2—S1—C1	108.47 (7)	C5—C6—H6	120.7
N1—S1—C1	105.54 (8)	C1—C6—H6	120.7
C7—N1—S1	127.02 (12)	N2—C7—N1	114.73 (14)
C7—N1—H1	119.6 (16)	N2—C7—C8	118.83 (15)
S1—N1—H1	110.9 (15)	N1—C7—C8	126.41 (15)
C11—N2—C7	123.13 (14)	C9—C8—C7	118.75 (16)
C11—N2—H2A	117.8 (14)	C9—C8—H8	120.6
C7—N2—H2A	118.9 (14)	C7—C8—H8	120.6
C2—C1—C6	122.01 (15)	C8—C9—C10	120.85 (16)
C2—C1—S1	118.68 (13)	C8—C9—H9	119.6
C6—C1—S1	119.21 (12)	C10—C9—H9	119.6
C1—C2—C3	118.62 (16)	C11—C10—C9	118.72 (16)
C1—C2—H2	120.7	C11—C10—H10	120.6
C3—C2—H2	120.7	C9—C10—H10	120.6
C4—C3—C2	119.91 (16)	N2—C11—C10	119.71 (16)
C4—C3—H3	120.0	N2—C11—H11	120.1
C2—C3—H3	120.0	C10—C11—H11	120.1
C3—C4—C5	120.73 (16)	O4—N3—O5	120.37 (13)
C3—C4—H4	119.6	O4—N3—O3	119.89 (13)
C5—C4—H4	119.6	O5—N3—O3	119.72 (13)
			
O1—S1—N1—C7	−171.95 (14)	C4—C5—C6—C1	−0.2 (2)
O2—S1—N1—C7	−42.97 (16)	C2—C1—C6—C5	0.9 (2)
C1—S1—N1—C7	73.37 (15)	S1—C1—C6—C5	177.27 (12)
O1—S1—C1—C2	114.78 (13)	C11—N2—C7—N1	−177.09 (13)
O2—S1—C1—C2	−17.97 (15)	C11—N2—C7—C8	1.2 (2)
N1—S1—C1—C2	−134.67 (13)	S1—N1—C7—N2	−164.75 (11)
O1—S1—C1—C6	−61.68 (14)	S1—N1—C7—C8	17.1 (2)
O2—S1—C1—C6	165.56 (12)	N2—C7—C8—C9	−1.3 (2)
N1—S1—C1—C6	48.86 (14)	N1—C7—C8—C9	176.77 (15)
C6—C1—C2—C3	−0.7 (2)	C7—C8—C9—C10	0.5 (3)
S1—C1—C2—C3	−177.06 (13)	C8—C9—C10—C11	0.4 (3)
C1—C2—C3—C4	−0.3 (3)	C7—N2—C11—C10	−0.2 (2)
C2—C3—C4—C5	1.0 (3)	C9—C10—C11—N2	−0.5 (2)
C3—C4—C5—C6	−0.8 (3)		

### Hydrogen-bond geometry (Å, °)

**Table:**

*D*—H···*A*	*D*—H	H···*A*	*D*···*A*	*D*—H···*A*
N1—H1···O5	0.86 (2)	1.91 (2)	2.760 (2)	171 (2)
N2—H2A···O3	0.91 (2)	1.84 (2)	2.7417 (18)	173 (2)
C8—H8···O2	0.95	2.38	3.009 (2)	123
C3—H3···O5^i^	0.95	2.52	3.432 (2)	162
C10—H10···O4^ii^	0.95	2.53	3.193 (2)	127
C11—H11···O3^iii^	0.95	2.56	3.434 (2)	153
C11—H11···O4^iii^	0.95	2.34	3.223 (2)	154

Symmetry codes: (i) −*x*+3/2, *y*+1/2, −*z*+1/2; (ii) *x*, *y*+1, *z*; (iii) −*x*, −*y*+1, −*z*.
